# Maternal adverse childhood experiences (ACEs) and their associations with intimate partner violence and child maltreatment: Results from a Brazilian birth cohort

**DOI:** 10.1016/j.ypmed.2021.106928

**Published:** 2021-12-23

**Authors:** Romina Buffarini, Gemma Hammerton, Carolina V.N. Coll, Suelen Cruz, Mariângela Freitas da Silveira, Joseph Murray

**Affiliations:** a Postgraduate Program in Epidemiology, Federal University of Pelotas, Pelotas, Brazil; b Population Health Sciences, Bristol Medical School, University of Bristol, Bristol, UK; c MRC Integrated Epidemiology Unit at the University of Bristol, Bristol, United Kingdom; d Human Development and Violence Research Centre, Federal University of Pelotas, Pelotas, Brazil

**Keywords:** Adverse childhood experiences, Women, Intimate partner violence, Child maltreatment, Cohort studies

## Abstract

Adverse childhood experiences (ACEs) have been found to predict many negative life outcomes. However, very little evidence exists on Intimate Partner Violence (IPV) and Child Maltreatment (CM). We investigated the impact of maternal ACEs on IPV and CM in three different: cumulative risk, individual adversities and particular groupings of ACEs. The 2015 Pelotas Birth Cohort, Southern Brazil, has followed a population-based sample mothers and children repeatedly until children were aged 4 years, when mothers provided data on ACEs, and current IPV and CM. ACEs were examined in three different ways: (i) as a cumulative risk score; (ii) individual adversities; and (iii) patterns of ACEs (Latent Class Analysis: LCA). One quarter (25.4%) of mothers reported having 5+ ACEs in childhood. Compared to mothers with no ACEs, those who reported 5+ ACEs, had 4.9 (95%CI 3.5; 6.7) times the risk of experiencing IPV and 3.8 (95%CI 2.5; 5.6) times the risk of reporting child maltreatment. LCA results also highlighted the major influence of multiple ACEs on later IPV and CM. However, individual ACEs related to violence (exposure to abuse or domestic violence) showed some specificity for both later IPV and CM, over and above the influence of cumulative childhood adversity. This is the first large study to demonstrate a strong link between maternal ACEs and both IPV and CM. Cumulative ACE exposure and some specificity in effects of childhood violence are important for later IPV and CM. Integrated prevention is essential for reducing the intergenerational transmission of adversity and violence.

## 1 Introduction

The profound and long lasting harmful consequences of specific experiences of child abuse and neglect have been documented in numerous studies for decades (Pechtel and Pizzagalli, 2011). In a now classic study of adult chronic disease and mortality, Felitti et al. (Felitti et al., 1998) demonstrated that cumulative exposure to a wide range of childhood adversities — including experiences such as parental mental illness, household drug abuse, and parental incarceration, as well as abuse and neglect — also had major effects on lifelong health outcomes. This wide range of adverse childhood experiences (ACEs) is understood to impose on biological and psychological stress systems of the growing child, with significant consequences for both physical and mental health, as well as risk behaviors into adult life. (Danese and McEwen, 2012; Pechtel and Pizzagalli, 2011).

An important systematic review of the health consequences of ACEs highlighted that future violence was one of the most significant outcomes of having experienced multiple ACEs in childhood (odds ratio > 7 in meta-analysis). (Hughes et al., 2017) However, primary studies located in this review did not investigate exposure to domestic violence as an outcome, instead using general outcomes such “being hit” in any context, (Bellis et al., 2020; Bellis et al., 2014a, 2014b; Bellis et al., 2014a, 2014b; Ford et al., 2016) “sexual violence” in any context, (Ports et al., 2016) or perpetration of physical violence against an intimate partner.(Anda et al., 2006). The latter study looking at perpetration of IPV found this was strongly associated with a history of multiple ACES, and also with difficulties controlling anger, both for women and men. Violence against women and children, which occurs primarily in the home, is a major human rights and public health issue, and a target for prevention in the UN’s Sustainable Development Goals for 2030.

Violence against women and children is driven by many complex social, cultural, community, family and individual-level determinants. Although there is a substantial literature on the intergenerational transmission of violence, including intimate partner violence (IPV) and child maltreatment (CM) (Assink et al., 2018; Kimber et al., 2018), there is little evidence on the influence of ACEs on violent victimization in intimate and family relationships from large, representative studies. We found no study of ACEs and child maltreatment in the next generation, and only two studies of ACEs and intimate partner violence victimization among adult women (IPV). (Nikulina et al., 2017; Randell et al., 2015). Both these studies used small and specific samples (*n* < 300, poor African American families, and college undergraduates) in the United States. Only in the study of poor African Americans was a significant association found, which had a very large magnitude (OR = 44 for those who had experienced ≥4 ACEs).

The ACEs literature has made very important contributions to understanding that individual childhood stressors rarely occur alone, and the accumulation of multiple stressors is particularly consequential for later life outcomes. However, the “cumulative ACE score” (the sum of the number of ACEs) that is normally considered in studies in this area, may hide nuances regarding particularly strong effects of some individual ACEs, and also the issue of whether different patterns of ACEs (certain combinations) may be more important than others. For this reason, recently, for outcomes of cardiovascular diseases,(Lacey et al., 2020) academic performance,(Merians et al., 2019) mental health, (Cavanaugh et al., 2015) substance use and incarceration,(Cavanaugh et al., 2015; Roos et al., 2016; Shin et al., 2018) studies have examined the effects of ACEs, considering not only cumulative ACE scores, but also each individual ACE experienced and, in person-centered analyses, the effects of different patterns of ACEs in the population. However, to date, research on ACEs and violence has not used this approach. New studies on ACEs and family violence, considering cumulative risk effects, individual types of ACEs, and different patterns of ACEs, can help identify those most at risk, and particular mechanisms that might be most appropriate to target in intervention.

The aim of this study was to examine the association between ACEs and IPV and CM in a large, population-based study, and compare the impact of cumulative ACEs, individual ACEs, and patterns of ACEs identified in person-based analyses. Considering the large evidence base on the intergenerational transmission of violence, (Assink et al., 2018; Ertem et al., 2000; Negriff, 2020) we hypothesized that among the various ACEs considered, childhood maltreatment might be most strongly associated with later IPV and CM.

## 2 Methods

### 2.1 Study design and population

Pelotas is a Southern Brazilian city with nearly 340,000 inhabitants. In 2015, we visited the five maternity hospitals in the city daily, and all deliveries (more than 99% of total births in the city) were identified. Newborns whose mothers were resident in the urban area of the city were eligible for the study, and information was obtained on 4275 (99%) of 4333 live births. Since then, participants have been assessed in regular follow-ups (at 3, 12 and 24 months and age 4 years). Information used in the current study was obtained from mothers (biological or social) at the last follow-up, when all cohort members were invited to participate in assessments at the research clinic (response rate 95.4%). During the visit, mothers answered standardizes computer-assisted questionnaires and both mothers and children had anthropometric measurements taken. The 2015 Cohort collected data on sociodemographic characteristics, health-related behaviors, neurocognitive development, and psychosocial information on children and their families. Detailed methodology of the cohort study methods has been published elsewhere. (Hallal et al., 2018).

Written informed consent was obtained from parents or guardians at each visit and ethical approval for the study was obtained from the Ethics Committee of the School of Physical Education, Federal University of Pelotas (CAAE registration number: 26746414.5.0000.5313); and psychosocial assessments assessed at age 4 years, including assessments of violence, were approved by the Ethics Committee of the Faculty of Medicine, Federal University of Pelotas (CAAE registration number: 03837318.6.0000.5317).

### 2.2 Measures

ACES. We evaluated 9 maternal ACEs, including all 7 from the original ACEs study by Felitti et al.(Felitti et al., 1998) (emotional abuse, physical abuse, sexual abuse, violence against household members, living with substance abusers, living with household members who were mentally ill or suicidal, living with household members who were imprisoned), as well as physical neglect and parental loss/divorce, which have been included in other studies of ACES. Mothers reported on these ACEs, experienced up to age 18, using a shortened version (19 items) of the World Health Organization (WHO) ACE-IQ questionnaire. (World Health Organization, 2018) The corresponding questions to each domain are presented in Appendix 1. Five questions had a yes/no answer response format, and 14 questions were rated on a 4-point scale (never, once, a few times and many times). ACEs were coded according to the binary version suggested by the WHO. (World Health Organization, 2018) If the participant scored positively (yes, or at least once) for any item in a domain, that domain was scored positively (ACE present); otherwise the ACEs was coded as absent. Then, all ACEs were summed, as is the convention, to produce a total ACEs score ranging from 0 to 9.

IPV was measured using the instrument of the Multi-country study on women’s health and violence against women (VAW) of the WHO. (Garcia-Moreno et al., 2005) The questionnaire assesses IPV in three domains: emotional (4 items), physical (6 items) and sexual (3 items), each item rated no/yes. All the items are summed to provide the total score (ranging from 0 to 13). Women who reported having experienced at least one act of IPV by a current or former intimate partner in the past 12 months scored positively (“yes”) for the outcome.

CM was assessed using the Juvenile Victimization Questionnaire, 2nd edition, Screener Sum Version, Caregiver Lifetime Form (JVQ-R2). (Finkelhor et al., 2005; Finkelhor and Ormrod, 2011) Lifetime victimization was based on the Aggregate Child Maltreatment, composed of five single items: physical abuse, emotional abuse, neglect, family abduction/custodial interference (included in the Child Maltreatment Module) and sexual assault by a known adult (measured in the Sexual Victimization Module). The child scored “yes” for CM when at least one of the five types of victimization was reported, whereas a “no” indicated no form of victimization.

Both IPV and CM were assessed in confidential interviews with mothers in the research centre. Trained female interviewers applied the questionnaires. Psychological support was available at the centre when positive responses indicating violence against the mother or the child were given. In these cases, the psychologists provided brief counselling and gave information about appropriate community support services. In cases of current risk of abuse the protocol was to report to social services.

### 2.3 Statistical analyses

Mothers with multiple births were analyzed only once by selecting only one child (the first-born sibling / first and second sibling in a case of triplets) for analysis (n excluded = 50). The analytic sample was restricted to mothers with ACEs and family violence data collected when children were aged 4 years and included 3712 participants for CM, and 3529 for IPV.

Analyses of the relationship between maternal ACES and family violence outcomes (IPV and CM) took three different approaches. The first used the traditional cumulative risk approach to analyse the relationship between the total ACEs score (scored 0, 1, 2, 3, 4, and 5 or more) and later violence. The second considered each individual ACE in relation to family violence outcomes. And the third person-based approach examined the relationship between patterns of exposure to ACES, identified using LCA, and later family violence.

LCA, used to identify different patterns of maternal ACEs, assumes that variability in response is due to a latent (unobserved) grouping. Latent classes were derived using individuals who had data present for at least one ACE (*N* = 3715) using robust full information maximum likelihood (FIML) estimation. Starting with a single class, a series of models were fitted, and theoretical and statistical steps were taken to decide on the optimal number of latent classes. Fit statistics included (a) the sample-size adjusted Bayesian information criterion (aBIC), (Schwarz, 1978) (b) the Bootstrap Likelihood Ratio Test (BLRT) (Nylund et al., 2007) and the Lo, Mendell & Rubin Likelihood Ratio Test (LMR-LRT) (Lo et al., 2001) which assesses the improvement in model fit for each additional class, and (c) bivariate model fit information—a test of the conditional independence assumption—using Pearson’s *χ*^2^. Model fit statistics for the 1-class to the 6-class model are shown in Appendix 2.

Maternal depression, severe anxiety, illicit drug use, and pregnancy at age 19 years or younger were used as validation criteria. Details of their measurement are shown in Appendix 3. Validation tests were based on the expectation that, compared to mothers with low ACEs, the other ACEs groups would show higher levels of mental illness, drug use and teenage pregnancy. To assess the associations between ACE groups and both validation criteria and study outcomes, a manual implementation of the bias-adjusted three-step method in M*plus* was applied. The logit parameters defining the relationship between modal and latent classes from the unconditional latent class model were used as constraints in the model including each validation outcome allowing prevalence ratios (PRs) and confidence intervals (CIs) for the associations to be calculated without influencing latent class membership.(Heron et al., 2015).

Poisson regression models with robust variance were run to compare the association between ACEs and IPV and CM, separately for each type of ACE variable (cumulative scores, individual ACEs, and latent classes). Prevalence ratios (PR) and 95% CI were calculated for each category of the exposure variables.(Barros and Hirakata, 2003) In order to assess possible variability in the associations according to maternal age, we tested for an interaction between the ACEs score (0 to 9) and maternal age (years). Presence of interaction would indicate that the prevalence ratios in the association between ACEs and violence (IPV or CM) are different according to maternal age.

All analyses involving the latent classes were carried out using M*plus* version 8.3 (Muthén and Muthén, 2009), and the remaining analyses were performed using STATA 16.1 (StataCorp, College Station, USA).

## 3 Results

ACEs were reported on by 3712 mothers, IPV by 3533, and CM by 3723, representing 86.8%, 82.6% and 87.1% of the original cohort respectively (*N* = 4275), respectively. Table 1 shows rates of maternal exposure to the individual ACEs as well as their cumulative ACE scores, and each family violence outcome (IPV and CM). A quarter of the mothers had experienced at least 5 ACEs; the mean number of ACEs was 3.0 (SD 2.1). The most common ACEs experienced by mothers were having a household member treated violently (62%), followed by parental separation or divorce and emotional abuse, which were each reported by about half of the women assessed. Physical abuse and parental alcohol and drug use were experienced by 39% and 35% of mothers, respectively. 22.8% of mothers experienced IPV during the past year, and 10.9% of children had experienced any form of maltreatment, according to maternal reports.

### 3.1 Cumulative maternal ACEs and family violence

Consistent with many ACE studies looking at the impact of multiple ACEs, we found a graded association between the number of ACEs and both family violence outcomes. For CM, the increased risk was strong for children whose mothers had experienced 3 or more ACEs, with especially strong risk for children of mothers who experienced 5+ ACEs compared with none (PR 3.8 95%CI 2.5;5.6) (Table 2). For IPV every additional ACE imparted greater risk of violence against women, and again for mothers who had experienced 5+ ACEs, the risk was very strong (PR 4.9 95%CI 3.5; 6.7). For both outcomes, no evidence of statistical interaction was found between ACEs and family violence outcomes, according to maternal age (*p* = 0.133 for CM and *p* = 0.257 for IPV); therefore the analyses are shown for the whole sample.

### 3.2 Individual maternal ACEs and their relationship with family violence

To consider whether individual ACEs have particularly strong effects, or whether a cumulative risk model captures all aspects of their association with family violence, next we analyzed each individual ACE separately. Crude results for individual ACEs showed that every individual adversity was associated with higher risk of CM and IPV, with the exception of parental death or separation or divorce. Several ACEs characterized by violence (household member treated violently, emotional abuse, physical abuse and contact sexual abuse) had particularly strong associations (PRs >2.0) with later IPV and CM. Notably, these remained associated with both outcomes even after adjustment for the total number of ACEs reported by mothers (Tables 3 and 4). In other words, experiencing individual violent adversities in childhood was important for later IPV and CM, over and above the overall context of adversity that the total ACE score represents.

### 3.3 Maternal ACEs grouped by Latent Class Analysis, and their association with family violence

**Fig. 1:**
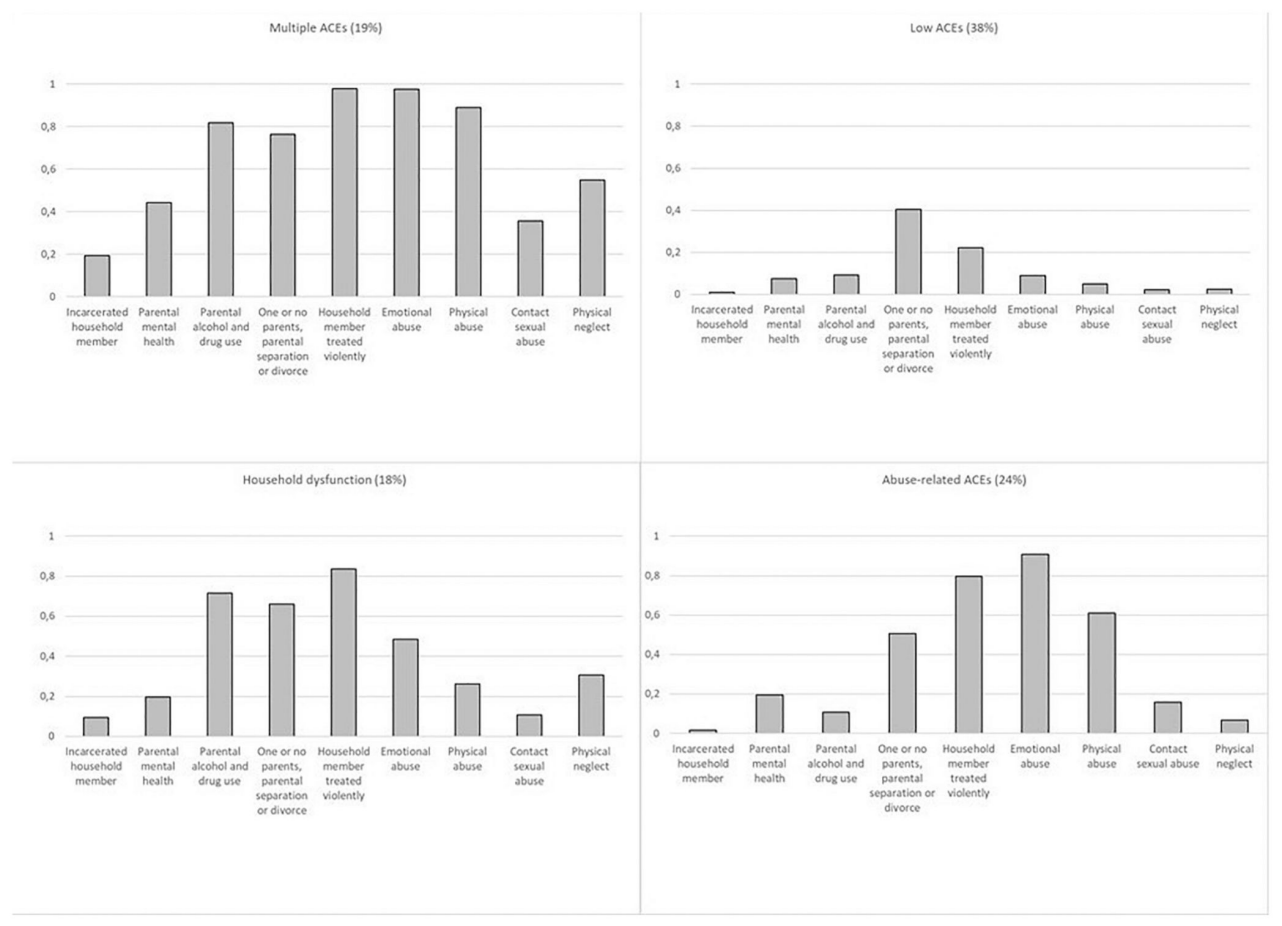
Within-class probabilities of each adverse childhood experience across the four latent classes of maternal adverse childhood experiences.

As well as considering the total ACE score and the significance of individual ACEs, we also examined how different patterns of exposure to ACEs might differentially impact family violence, using Latent Class Analysis (LCA). The LCA produced a four-class solution, shown in Fig. 1. The Figure shows the probabilities of reporting each of the 9 ACEs, for each class. We gave names to these classes according to the patterns of adversity most common in each. Nineteen percent of the mothers were grouped in the “multiple ACEs” class - showing the highest probabilities of experiencing all the ACEs examined. 38% were grouped in a ‘low ACEs’ class; 18% in the class called “household dysfunction” (characterized mainly by ACEs such as parental alcohol and drug use, but also including some violence), and 24% were grouped in the ‘abuse-related ACEs’ class. This class was well separated from the ‘multiple ACEs’ by its high endorsements of the violence experience items (witnessing intra parental abuse, emotional abuse, physical abuse) and low endorsements of other items.

Finally, we examined family violence outcomes comparing the ‘low ACES’ class with each of the other ACE classes. ‘Household dysfunction’ and ‘abuse-related ACEs’ classes were associated with CM and IPV in similar magnitudes – with PRs around 2. Women in the ‘multiple ACEs’ class had 5 times higher risk of reporting CM (PR: 5.0 95%CI 3.6; 6.8) and 4.3 times higher risk of experiencing IPV (PR 4.3 95%CI 3.5; 5.3), compared with women belonging to the ‘low ACEs’ class.

One can compare the increased risk for family violence imparted by being in the “multiple ACEs” latent class (vs. the “low ACEs” latent class), and the risk imparted by having 5+ ACEs (v.s having none) in our initial analyses of a simple count of ACEs (shown in Table 2). For both IPV and CM, the risk for mothers with 5+ ACEs (Table 2) was similar to risk imparted by being in the ‘multiple ACEs’ class compared to the ‘low ACEs’ class (Table 5).

## 4 Discussion

In this this large, population-based study, risk for both IPV and CM was about 5 times larger for women who had experienced 5 or more ACEs compared to none. The increased risk following the experience of multiple ACEs was also confirmed in Latent Class Analyses of ACEs. However, we also identified some individual ACEs that carried unique risk effects: mothers who had witnessed or suffered abuse in childhood had particularly elevated risk for later IPV and CM, even after adjusting for the cumulative number of ACEs experienced. In a nutshell, cumulative childhood adversity has a large impact on risk for later IPV and CM, but there is also some additional, specific influence of childhood abuse and witnessing of violence.

A systematic review previously showed that violence was one of the most important health-related outcomes of multiple ACEs. (Hughes et al., 2017) However, few surveys have examined CM or IPV against women in relation to ACEs. IPV and CM are major public health problems, as well as human rights violations, associated with numerous physical, mental, and social difficulties through the life-course. (Campbell, 2002; Gilbert et al., 2009). The population-based evidence presented in the current study, showing that multiple ACEs have a strong influence on IPV and CM, thus demonstrates new and important burdens of ACEs for women, children and health systems. Although many con-current factors have been identified that increase risk for IPV and CM, this long reach of childhood disadvantage into the families of the next generation is a critical issue for prevention.

The strong influence of ACEs on later family violence may be explained by the multiple psychosocial consequences of ACEs throughout life, including poverty, mental illness, and substance use (Alvarez et al., 2019; Ashton et al., 2016; Bellis et al., 2020; Cavanaugh et al., 2015; Hughes et al., 2017), which themselves may put women and children at risk for domestic violence. In our cohort, we previously reported that IPV and CM (and their co-occurrence) arise in the context of concentrated psychosocial disadvantage, including neighborhood violence, low income, paternal antisocial behaviour, poor mother-partner relationship, maternal depression, and younger maternal age. (Buffarini et al., 2021) Thus, intergenerational continuity in social disadvantage may be a key mechanism between multiple ACEs and later IPV and CM.

Understanding the unique contribution of abuse and the witnessing of household violence for IPV and CM (over and above the effect of experiencing multiple ACEs), requires considering other, more specific mechanisms involved in the intergenerational transmission of violence. There is already a long history of research on the intergenerational transmission of CM (Assink et al., 2018), however, ours is the first study we are aware of to demonstrate this transmission in the context of cumulative ACEs – contexts in which childhood abuse and witnessing violence often occur. One main theory for the intergenerational transmission of violence is that social learning processes involved in the experience of childhood abuse and the witnessing of domestic violence transmit across the life-course to affect involvement with antisocial partners and exposure to their violence (Abramsky et al., 2011; Ehrensaft et al., 2003; Jouriles et al., 2001; Whitfield et al., 2003). The social learning model implies that child exposure to violence in the household could lead women that to view violence and coercive norms as acceptable or effective means of resolving conflicts with partners and/or children.(Jouriles et al., 2001) These processes thus could increase risk for later IPV or CM, over and above the influence of other childhood adversities.

Maternal exposure to ACEs was extremely high in the current study – about one quarter of mothers reported having experienced 5+ ACEs. In two other populations, 5+ ACEs have been reported with prevalence rates of 4% and 9% (Merians et al., 2019; Pirkola et al., 2005; Ye and Reyes-Salvail, 2014), and 4+ ACEs have been reported with rates of between 9% and 20% (Ashton et al., 2016; Lacey et al., 2020; Ramiro et al., 2010). The current study was conducted in a relatively poor city of Southern Brazil, where socioeconomic conditions were considerably worse when mothers were in their childhood compared to today. Comparisons of cohorts in the same city over the last 30 years has shown significant improvement in terms of maternal schooling, household crowding, treated water, assets, and inequalities of these indicators through time.(Bertoldi et al., 2019) This may speak to children growing up in the same society today experiencing fewer economically induced ACEs. However, despite such material advances, violence in Brazil as a whole, and in Pelotas city, has increased dramatically over previous decades,(Murray et al., 2013) and children still suffer severe levels of child maltreatment, (Viola et al., 2016) pointing to a major need for preventive interventions.

The findings from the current study, together with those from many others on the intergenerational transmission of violence, speak to the importance of a life-course approach toward prevention of violence against women and children, and the need for integrated intervention approaches at the family level, as well as policies that address distal social and cultural causes. Parents/caregivers are the primary socialization agents involved in children’s care and development, and interventions with them aiming to decrease child maltreatment could both protect children now and reduce family violence in the future.(World Health Organization, 2016) For example, nurse home visiting programs have been associated with reductions in both child maltreatment and children’s own antisocial behaviour many years later.(Olds et al., 1998) Further, conflict resolution training for parents may reduce interparental violence, and thus also children’ s exposure to violence in the home.(Ellsberg et al., 2015) Because IPV and CM frequently overlap, interventions at the family level may succeed in reducing both forms of violence.(World Health Organization, 2016) There is need to include both males and females in IPV prevention efforts. Although not the focus of analysis of the current study, paternal antisocial behaviour is known to increase the risk for both IPV, CM, and their co-occurrence,(Buffarini et al., 2021) and many prevention programmes, including parenting programmes, child social skills training, cognitive behavioural interventions, have shown effective in reducing male antisocial behaviour. (Farrington and Welsh, 2006).

This study has important strengths, including a population-based study design with a large sample and a high follow-up rate, use of validated and widely used instruments, and excellent responses rate for the questionnaires on ACEs and both outcomes. The use of a LCA approach to considering exposure to ACEs has interesting benefits over traditional sum scores which have been criticized regarding psychometric properties and other issues.(Afifi et al., 2020; McLennan et al., 2020) Notably, few mothers in the current study experienced zero ACEs, a common reference category used in the literature; the “low ACE” group identified in LCA potentially provides a more meaningful comparison group. Limitations of our study include the fact that the mother was the single informant for ACEs and violence outcomes, raising the possibility of common reporter and social desirability biases, that could result in overestimated associations. A second limitation is that, as with nearly all studies of ACEs, maternal ACEs were retrospectively reported and this information may be biased by impaired memory or psychological functioning. We did not have information about perpetrators of IPV or CM. Lastly, we lack measures related to maternal socioeconomic conditions and other environmental variables from the time of mothers’ childhoods, as well as genetic data, that might represent confounding factors. Of course, the study results cannot be generalized to other populations without replication, even within the large country of Brazil, with considerable social, economic and cultural variability.

## 5 Conclusion

Our findings from this population-based birth cohort study show a large increase in risk of IPV and CM linked to multiple maternal ACEs, as well as specific exposures to violence in women’s childhoods. Integrated intervention approaches at the family level are needed to break the cycle of violence and improve the lives of future generations.

## Supplementary Material

Supplementary material

## Figures and Tables

**Table 1:** Description of the study sample.

Individual maternal ACEs	% (95% CI)
Parental alcohol and drug use	34.9 (33.4; 36.5)
Parental mental health problems	19.7 (18.4; 21.0)
Incarcerated household member	6.2 (5.5; 7.0)
One or no parents, parental separation or divorce	54.5 (52.9; 56.1)
Household member treated violently	62.0 (60.4; 63.5)
Emotional abuse	53.2 (51.6; 54.8)
Physical abuse	38.6 (37.1; 40.2)
Contact sexual abuse	13.5 (12.4; 14.6)
Physical neglect	18.7 (17.5; 20.0)
ACEs score	
0	12.9
1	16.8
2	15.3
3	15.4
4	14.4
5+	25.4
Outcomes	
Intimate partner violence	22.8 (21.4; 24.2)
Child maltreatment	10.9 (10.0; 11.9)

ACE(s) = Adverse Childhood Experience(s), N = 3712. Intimate partner violence, *N* = 3533; Child maltreatment, *N* = 3723.

**Table 2:** Crude associations between maternal adverse childhood experiences (ACEs) cumulative score and the child maltreatment (CM) and intimate partner violence (IPV) at 4 years old; *N CM = 3712; N IPV = 3529*.

ACEs score	CM	IPV
	PR (95% CI)	PR (95% CI)
0	Reference	Reference
1	1.2 (0.7; 1.9)	1.5 (1.0; 2.2)
2	1.0 (0.6; 1.6)	2.5 (1.7; 3.5)
3	1.9 (1.2; 3.1)	2.2 (1.5; 3.1)
4	2.4 (1.5; 3.7)	3.6 (2.5; 5.0)
5 or more	3.8 (2.5; 5.6)	4.9 (3.5; 6.7)

*p* < 0.001 for both outcomes.

**Table 3:** Crude and adjusted associations between individual maternal adverse childhood experiences (ACEs) and child maltreatment (CM); *N* = *3712*.

Individual ACEs	Crude		Adjusted	
	PR (95% CI)	*p*-value	PR (95% CI)	p-value
Parental alcohol and drug use	1.6 (1.3; 1.9)	<0.001	1.0 (0.8; 1.2)	0.663
Parental mental health problems	2.0 (1.7; 2.5)	<0.001	1.5 (1.2; 1.8)	<0.001
Incarcerated household member	1.9 (1.4; 2.5)	<0.001	1.2 (0.9; 1.5)	0.309
One or no parents, parental separation or divorce	1.2 (1.0; 1.4)	0.084	0.9 (0.7; 1.1)	0.149
Household member treated violently	2.6 (2.1; 3.4)	<0.001	1.7 (1.3; 2.2)	<0.001
Emotional abuse	2.3 (1.9; 2.9)	<0.001	1.5 (1.2; 1.8)	0.001
Physical abuse	2.4 (2.0; 2.9)	<0.001	1.6 (1.3; 2.0)	<0.001
Contact sexual abuse	2.3 (1.9; 2.8)	<0.001	1.5 (1.2; 1.9)	<0.001
Physical neglect	2.0 (1.7; 2.5)	<0.001	1.3 (1.0; 1.6)	0.018

Adjusted for number of ACEs (in each regression the exposure ACE was removed from the score).

**Table 4:** Crude and adjusted associations between individual maternal adverse childhood experiences (ACEs) and intimate partner violence (IPV); *N* = *3529*.

Individual ACEs	Crude		Adjusted	
	PR (95% CI)	p-value	PR (95% CI)	p-value
Parental alcohol and drug use	1.7 (1.5; 2.0)	<0.001	1.1 (1.0; 1.3)	0.042
Parental mental health problems	1.5 (1.3; 1.7)	<0.001	1.1 (0.9; 1.2)	0.267
Incarcerated household member	1.7 (1.4; 2.0)	<0.001	1.1 (0.9; 1.3)	0.406
One or no parents, parental separation or divorce	1.5 (1.3; 1.7)	0.084	1.1 (1.0; 1.3)	0.058
Household member treated violently	2.3 (2.0; 2.6)	<0.001	1.5 (1.2; 1.7)	<0.001
Emotional abuse	2.4 (2.1; 2.8)	<0.001	1.7 (1.4; 2.0)	<0.001
Physical abuse	2.0 (1.8; 2.2)	<0.001	1.3 (1.1; 1.5)	<0.001
Contact sexual abuse	2.1 (1.8; 2.3)	<0.001	1.4 (1.3; 1.7)	<0.001
Physical neglect	1.8 (1.6; 2.0)	<0.001	1.1 (1.0; 1.3)	0.068

Adjusted for number of ACEs (in each regression the exposure ACE was removed from the score).

**Table 5:** Crude associations between latent classes of maternal adverse childhood experiences (ACEs) and the violence outcomes when offspring are age 4-year (child maltreatment (CM) and intimate partner violence (IPV)); *N CM = 3712; N IPV = 3529*.

Latent classes of ACEs	CM	IPV
	PR (95% CI)	PR (95% CI)
Low ACEs	Reference	Reference
Household dysfunction	2.1 (1.2, 3.4)	2.3 (1.7, 3.1)
Abuse-related ACEs	2.5 (1.7, 3.7)	2.4 (1.9, 3.1)
Multiple ACEs	5.0 (3.6, 6.8)	4.3 (3.5, 5.3)

p < 0.001 for both outcomes.
