# Supplementary material for: Maternal adverse childhood experiences (ACEs) and their associations with intimate partner violence and child maltreatment: Results from a Brazilian birth cohort
Source: Prev Med. Author manuscript; Available in PMC 2023 Aug 9. (PMC7614899; doi:10.1016/j.ypmed.2021.106928)
Supplement: Supplementary material [file EMS182172-supplement-Supplementary_material.docx]

# Appendices

**Appendix 1. Questions of WHO ACE-IQ applied in the 2015 Pelotas Birth Cohort**

**Calculating the ACEs score from the ACE-IQ - BINARY VERSION**

| **Category** | **Question** |
| --- | --- |
| **Alcohol and/or drug abuser in the household** | Did you live with a household member who was a problem drinker or alcoholic, or misused street or prescription drugs? |
| **Someone chronically depressed, mentally ill, institutionalized or suicidal** | Did you live with a household member who was depressed, mentally ill or suicidal? |
| **Incarcerated household member** | Did you live with a household member who was ever sent to jail or prison? |
| **One or no parents, parental separation or divorce** | Were your parents ever separated or divorced?  **OR**  Did your mother, father or guardian die? |
| **Household member treated violently** | Did you see or hear a parent or other person in your home (adult or child) being yelled at, screamed at, sworn at, insulted or humiliated?  **OR**  Did you see or hear a parent or household member in your home being slapped, kicked, punched or beaten up?  **OR**  Did you see or hear a parent or household member in your home being hit or cut with an object, such as a stick (or cane), bottle, club, knife, whip etc.? |
| **Emotional abuse** | Did a parent, guardian or other household member yell, scream or swear at you, insult or humiliate you?  **OR**  Did a parent, guardian or other household member threaten to, or actually, abandon you or throw you out of the house? |
| **Physical abuse** | Did a parent, guardian or other household member spank, slap, kick, punch or beat you up?  **OR**  Did a parent, guardian or other household member hit or cut you with an object, such as a stick (or cane), bottle, club, knife, whip etc.? |
| **Contact sexual abuse** | Did someone touch or fondle you in a sexual way when you did not want them to?  **OR**  Did someone make you touch their body in a sexual way when you did not want them to?  **OR**  Did someone attempt oral, anal, or vaginal intercourse with you when you did not want them to?  **OR**  Did someone actually have oral, anal, or vaginal intercourse with you when you did not want them to? |
| **Physical neglect** | How often did your parents/guardians not give you enough food even when they could easily have done so?  **OR**  How often were your parents/guardians too drunk or intoxicated by drugs to take care of you?  **OR**  How often did our parents/guardians not send you to school even when it was available.? |

**Appendix 2. Fit statistics and decision on latent classes**

Both the four- and five-class models provided a good fit to the data. Although the aBIC was lower for the four-class model (indicating better fit), the BLRT and LMR-LRT suggested that the five-class model was an improvement in fit over the four-class model. Both the four- and five-class models were therefore investigated further. The four-class model included classes that were both qualitatively distinct and more theoretically meaningful, therefore this model was taken forwards for all further analysis.

**Supplementary Table 1**. Fit statistics for latent classes of maternal adverse childhood experiences; *N* = 3716

| Classes | Parameters | Likelihood | aBIC | Entropy | Bivariate fit | BLRT | LMR-LRT |
| --- | --- | --- | --- | --- | --- | --- | --- |
| 1 | 9 | -18433 | 36911 | n/a | 7151 | *p* < 0.001 | *p* < 0.001 |
| 2 | 19 | -16607 | 33310 | 0.74 | 411 | *p* < 0.001 | *p* < 0.001 |
| 3 | 29 | -16410 | 32967 | 0.69 | 100 | *p* < 0.001 | *p* < 0.001 |
| **4** | **39** | **-16312** | **32821** | **0.65** | **36** | ***p* < 0.001** | ***p* < 0.001** |
| 5 | 49 | -16291 | 32829 | 0.70 | 25 | *p* < 0.001 | *p* = 0.014 |
| 6 | 59 | -16278 | 32854 | 0.62 | 18 | *p* = 0.140 | *p* = 0.361 |

aBIC: sample-size adjusted Bayesian information criterion (lower values indicate preferred models); BLRT: Bootstrap Likelihood Ratio Test; LMR-LRT: Lo, Mendell & Rubin Likelihood Ratio Test (high values indicate no evidence of improvement in fit from model with one less class)

**Appendix 3. Validation outcomes**

Maternal depression, severe anxiety, illicit drug use, and pregnancy at age 19 years or younger were used as validation criteria, with the expectation that, compared to the low ACEs group, the other ACEs groups would show higher levels of mental illness, drug use and teenage pregnancy. Maternal depression was defined as scoring 13 or more points on the Edinburgh Postnatal Depression Scale (EPDS), applied at child age 3-months, and previously validated in the same population. Maternal anxiety was assessed using the GAD-7 at child age 12 months and dichotomised to represent severe levels of anxiety. Maternal drug use was assessed at child age 48-months with the Alcohol, Smoking and Substance Involvement Screening Test (ASSIST), and characterised as use of any illicit substance in the three months prior to interview. Teenage pregnancy was defined as pregnancy at age 19 years or younger. Univariable associations between the latent classes of ACEs and each of these validation criteria are shown in Supplementary Table 2.

**Supplementary Table 2** - Univariable associations between the latent classes of maternal adverse childhood experiences (ACEs) and maternal depression, severe anxiety, illicit drug use and pregnancy at age 19 years or younger; showing prevalence ratio (95% confidence interval); *Ns* vary across models from 3589 to 3715.

|  | **Latent classes of maternal adverse childhood experiences (ACEs)** | | | | |
| --- | --- | --- | --- | --- | --- |
| **Validation outcomes** | Low ACEs | Household dysfunction ACEs | Abuse-related ACEs | High ACEs | *p value* |
| Maternal depression (11%) | Ref. | 1.93 (1.26, 2.94) | 1.49 (1.02, 2.18) | 2.92 (2.19, 3.91) | < 0.001 |
| Maternal severe anxiety (12%) | Ref. | 2.37 (1.49, 3.78) | 2.62 (1.81, 3.81) | 4.63 (3.39, 6.32) | < 0.001 |
| Maternal illicit drug use (4%) | Ref. | 2.30 (0.94, 5.62) | 2.33 (1.12, 4.82) | 5.14 (2.88, 9.16) | < 0.001 |
| Pregnancy ≤ 19 years (14%) | Ref. | 1.86 (1.41, 2.47) | 0.87 (0.62, 1.20) | 1.24 (0.95, 1.62) | < 0.001 |
